# Competency-Based Postgraduate Medical Education: Past, Present and Future

**DOI:** 10.3205/zma001146

**Published:** 2017-11-15

**Authors:** Olle ten Cate

**Affiliations:** 1 University Medical Center Utrecht, Center for Research and Development of Education, Utrecht, The Netherlands

**Keywords:** Competency-based medical education (CBME), competency, competence, CanMEDS, entrustable professional activities, milestones

## Abstract

Since the turn of the twenty-first century, competency-based medical education (CBME) has become a dominant approach to postgraduate medical education in many countries. CBME has a history dating back half a century and is rooted in general educational approaches such as outcome-based education and mastery learning. Despite controversies around the terminology and the CBME approach, important national medical regulatory bodies in Canada, the United States, and other countries have embraced CBME. CBME can be characterized as having two distinct features: a focus on specific domains of competence, and a relative independence of time in training, making it an individualized approach that is particularly applicable in workplace training. It is not the length of training that determines a person’s readiness for unsupervised practice, but the attained competence or competencies. This shift in focus makes CBME different from traditional training. In this contribution, definitions of CBME and related concepts are detailed.

## Zusammenfassung

Seit Beginn des 21. Jahrhunderts ist die kompetenzbasierte medizinische Weiterbildung (Competency-Based Medical Education, CBME) zum dominanten pädagogischen Ansatz in der medizinischen Ausbildung vieler Länder geworden. Mit einer Geschichte, die ein halbes Jahrhundert zurückgeht, ist die CBME in pädagogischen Ansätzen wie ergebnisorientierter Ausbildung und „Mastery Learning“ verwurzelt. Trotz der Kontroversen um die Terminologie und den CBME-Ansatz haben wichtige nationale medizinische Aufsichtsbehörden in Kanada, den Vereinigten Staaten und in anderen Ländern eine Bereitschaft für CBME gezeigt. CBME kann durch zwei deutliche Merkmale charakterisiert werden: durch einen Fokus auf spezifische Kompetenzbereiche und durch eine relative Unabhängigkeit vom Zeitpunkt innerhalb der Ausbildung; so wird CBME zu einem individualisierten Ansatz, der besonders beim Lernen am Arbeitsplatz anwendbar ist. Es ist nicht die Länge der Ausbildung, die das Bereitsein des Einzelnen für nicht supervidierte Praxis bestimmt, sondern die gewonnene Kompetenz oder die gewonnenen Kompetenzen. Diese Schwerpunktverlagerung unterscheidet CBME von der traditionellen Ausbildung. In diesem Beitrag werden Definitionen von CBME und verwandten Konzepten ausgeführt.

## Introduction

Competency-based medical education (CBME) or training (CBMT) has become widely used terminology since the turn of the twenty-first century. Despite its ubiquitous use, there is variation in the use of the terminology and related concepts. In this entry a brief historical overview of the concept is provided, followed by a focus on a clear justification and definition of CBME, competence, competency, and closely related concepts. 

## History

In 1949, long before the term “competency-based” education was being used in medical or other areas of education, educational psychologist Ralph Tyler sowed its first seeds in what has become known as the “Tyler rationale” [1]. He posed four powerful questions any education institution should address:

1. What purposes should a school seek to attain?
2. What educational experien¬ces can be provided to attain these purposes?
3. How can these be organized?
4. How can one determine whether these purposes are being attained?

This outcome-based thinking of education differed from education practice before. Since then, many educationalists have expanded on his ideas, most prominently Benjamin Bloom, whose *taxonomy of educational objectives*, including a cognitive (knowledge), a psychomotor (manual skills), and an affective (attitudes) domain, has dominated most of the world’s thinking of educational objectives [2]. The significance of these contributions was that education became more systematically focused on predefined outcomes than on evolved tradition. In 1963 Carroll observed that, given equivalent learning time, students with different aptitudes diverge in their learning performance; some do not attain the required performance goal [3]. To avoid variable outcome of education, he said, each learner must be allowed the learning time he or she needs to attain a specific learning goal. This view revolutionized the educational thinking by recognizing that a similar mastery of skills requires flexibility and individualization. The focus on outcomes led to approaches such as Bloom’s “personalized systems of instruction” and “mastery learning” to ensure that as many students in a class as possible meet a required learning criterion [4]. Several studies have illustrated its success and in many countries the relationship between education and future workplaces became tighter [5]. Vocational education and training became more an instrument of economic forces, as influential people outside education started formulating aims and content for it, to ensure that workers would be productive. The vast technological and scientific changes and globalization since the 1980s, with education lagging behind, led schools to introduce *employment competencies*, justified by the wish to increase levels of skills and flexibility to serve a competitive economy. At the university level these reforms were not always welcomed, as it was feared that a heavier weight of industry needs could hamper general academic education. The very nature of liberal arts – the freedom of academic development – is not really compatible with the strong utilitarian nature of industry-determined outcomes. 

### Competency-based medical education

Before the massive expansion of postgraduate training, Case Western Reserve University’s medical school in Cleveland, Ohio was among the first to recognize, as early as the 1950s, that the content of medical training would be more efficiently delivered if focused on clinical relevance, next to the systematic, scientific foundations of individual disciplines. With Ralph Tyler as a consultant, this school integrated pre-clinical courses with clinically relevant objectives, to make the transition from theory to practice more natural [6]. It was a first step toward outcome-based medical education, the precursor of competency-based medical education. This outcome direction was adopted by numerous schools, particularly in the Western world, from the 1960s until the present day [7]. 

Medical education and teacher education – on one hand both academic disciplines, and on the other hand both directed toward a professional vocation – were among the first to advocate competency-based education. An excellent early description of competency-based medical education was coined by McGaghie and colleagues in 1978. The authors distinguish CBME from subject-oriented and integrated curricula by

1. its organization around *functions required for the practice* of medicine in a specified setting,
2. the conviction that *all medical students can master* the basic performance objectives, and
3. the justification that learning and learning processes *can then be empirically tested*.

“The intended outcome [of CBME] is a health-professional who can practice medicine at a defined level of proficiency, in accord with local conditions, to meet local needs” [8].

#### Competency-based postgraduate medical education

As competency-based medical education is outcome-based, a focus of CBME on postgraduate training has been dominant. In western countries, unsupervised practice of healthcare, the dominant outcome of the training of physicians, is almost exclusively the prerogative of medical specialists after postgraduate training, which now includes primary care. 

Competency-based (postgraduate) medical education is now a widely used terminology, especially after the introduction of the *CanMEDS* framework (Canadian Medical Education Directives for Specialists) project in the 1990s [9], followed by the Outcome Project of the ACGME (Accreditation Council for Graduate Medical Education in the USA) [10], [11]. The CBME movement has met with criticism, part of which can be attributed to varying interpretations of what it is, and part to the way it is being applied [12], [13], [14].

## Definitions

Many authors have attempted to clarify the “fuzzy” concepts of competence and competency. Multidimensional typologies of competence have been described, one of which distinguishes a conceptual–operational axis versus a personal–occupational axis. Medical competence would be situated primarily in the functional quadrant of this general typology, being both operational and occupational. But many other dimensions have been discussed extensively in the literature, such as context-free versus context-specific, knowledge versus capability, behavior versus ability, learnable versus unchangeable, performance-oriented versus development-oriented.

The medical education community has also defined competence in many different ways [15]. A recent authoritative definition captures what the majority of medical educators would probably agree with: “The habitual and judicious use of communication, knowledge, technical skills, clinical reasoning, emotions, values, and reflection in daily practice for the benefit of the individual and community being served” [16]. This definition aims to comprehensively encompass all elements of professional medical functioning and should be used as a singular noun without article (i.e., not a competence). Following this definition, “competences,” in the plural, is not useful terminology. As “competencies” is considered linguistically synonymous to “competences” [17], we shall use “competencies” as the word for parts that together constitute the full spectrum of medical competence.

The word “competency,” formulated most literally as “the ability to do something successfully or efficiently” [17], has led to confusion among educators. As competency-based education did not always lived up to its promise, the concept has been redefined often. The Educational Council of the Netherlands proposed a useful literature-derived definition of competency that includes six features: a competency is *specific, integrative, durable, focused on performance, learnable*, and *competencies are mutually dependent* [18]. This accords with a more recent definition by Albanese and colleagues, who add that competencies should reflect external expectations and should lead to *behavior that is measurable using absolute standards*, that is, independent of other learners [19]. Other authors have stressed that the ability to act successfully is to some extent context dependent. A person can possess a competency in one context, for example during the day in a well-equipped hospital, but not in a different context, for example during the night in a remote rural area with little medical support. If the ability to perform well in the full scope of the medical profession equates with “medical competence,” then *a medical competency* can thus be defined as a l*earnable, durable, and measurable ability to execute a specific, integrative task that is a part of the full range of tasks that constitute the medical profession. It is a generalized ability that may vary somewhat, depending on the context*. Following this definition, neither the general entities of the CanMEDS framework nor those of the ACGME framework should be called “competencies.” The seven CanMEDs units are rightfully designated as “roles” (medical expert, communicator, collaborator, leader, scholar, health advocate, professional) [20], in contrast with the six ACGME descriptors (patient care, medical knowledge, interpersonal and communication skills, practice-based learning and improvement, system-based practice, professionalism), which have initially been named “core competencies” [10]. If “competence” is the broad quality of the physician as defined by Epstein and Hundert, then such general elements of competency frameworks are best designated as *“domains of competence”.* Domains of competence are broad entities that include multiple competencies. For example, the domain of patient care would include competencies such as the ability to “gather information about the patient,” “perform an accurate physical examination,” and “develop and carry out a management plan.” This terminology has been supported by Englander and colleagues [21]. 

The adjective “competent” describes a person who has “the ability to do something,” or a “competency”. “Competent” also has the connotation of a legal right to act or judge. The authorization to judge or act can be considered dependent on the demonstration of sufficient mastery of a competency. In this sense, a competent person can act, but also has an *authority or right* to act, in the sense that unqualified persons do not have this right [17]. This is a relevant addition for professionals with a legal responsibility, among whom are medical specialists. Their license provides rights and duties, bound to their competence.

“Competency-based medical education” evolves from its founding concepts of competency and competence. Linguistically, “competency-based education” is not fully logical, as it appears to refer to education that is *based on* competencies rather than producing them. Other languages use “competency-directed” or “competency-oriented,” but we will stick to the common usage. Based on a literature review, Frank and colleagues state that CBME is “an approach to preparing physicians for practice that is fundamentally oriented to graduate outcome abilities and the organization around competencies derived from an analysis of societal and patient needs. It de-emphasizes time-based training and promises a greater accountability, flexibility, and learner-centeredness” [22]. While this is strictly not a definition but rather a circumscription, it includes a new element that distinguishes CBME programs from other programs: time independence. This is indeed fundamental to CBME, which can be argued for different reasons [23]. If competency-based education focuses on certifying or graduating students as soon as they are competent, time in training loses some of its relevance. Theoretically, residents who start education on a high level of capability and prior experience should arrive at a predefined level of competence earlier than those who start with little experience. Education in settings that are workplaces instead of classes is already highly individualized. Given the natural difference in workplaces, learning experiences will be different too. This brings us to two defining features of competency-based medical education:

1. its focus on outcomes formulated as specific competencies, and
2. its independence of the length of time in training. Competence-dependent certification instead of time-dependent certification is reminiscent of Bloom’s mastery learning.

Given the definitions of “competence,” “competency,” and “competent” for educational purposes as delineated above, competency-based medical education can thus be defined as: *Education for the medical profession that is targeted at a fixed level of proficiency in one or more medical competencies.* The individualized and time-independent nature of CBME stems naturally from this definition, as education is finished when a pre-set level of competence is reached, rather than after a fixed number of years. In this definition CBME is not restricted to workplace learning, but in practice the approach is specifically useful in settings that allow for individualized learning and flexibility such as the clinical workplace. The additions and descriptions, added by Frank et al [22], such as the societal origin of the competencies and its learner centeredness, are useful and defendable, but linguistically not necessary to be included in the definition.

## Collateral Definitions

Related to competency-based medical education, a number of other concepts have been used which are valuable to include here. The design of competency frameworks, such as CanMEDS and the ACGME framework, has resulted in detailed descriptions of the qualities trainees must show. Domains of competence have been analytically described, with sub-competencies, key competencies, core competencies, and enabling competencies [9], [10] to operationalize the rather broad domains into manageable units for teaching and assessment, and to translate them into regulations. However, in doing so, such analytic descriptions tend to become theoretical, context independent, and to move away from practice, and from the practical definition of competency that the Concise Oxford English Dictionary provides: to do something successfully [17]. We recommend that these subdomains of competence are not called competencies, as they usually do not accord with the definition of competency (see above), and they cannot easily be “attained” or measured in a valid way, specifically those domains outside medical-technical skills [24]. For example, “ethical conduct toward patients” is an important quality, but rather a prerequisite for circumscriptive tasks than a competency in itself. In several competency frameworks many such “competencies” have a rather theoretical nature. 

In 2005, the term “entrustable professional activity” (EPA) was introduced to reconnect competency frameworks to the workplace [25]. An EPA is “a unit of professional practice, defined as a task or responsibility to be entrusted to a trainee once sufficient specific competence is reached to allow for unsupervised practice. EPAs are independently executable within a time frame, observable and measurable in their process and outcome, and suitable for entrustment decisions.” The capability to execute an EPA can be considered a competency, as defined earlier. Working with EPAs has been called a synthetic or holistic approach, as it brings together multiple domains of competence into relevant tasks of the profession [26]. The essence of “trusting” a trainee, translated to “entrustment decisions” about EPAs, counters the notion of a check-box approach of CBME that has been said to reduce the medical profession to a series of superficial skills [27]. The full description of an EPA includes the connection with a competency framework [28]. When evaluating learners with a focus on the question “How much supervision does this learner with this EPA require?” [29], [30], [31], then the competencies that underpin its answer may be rather called *facets of competence*, which is actually a better wording than competencies [32]. Scales that signify level of supervision for entrustment decisions as now being called *entrustablility scales* [31], [33].

EPAs have been proposed in a wide range of specialty programs, including pediatrics, psychiatry, internal medicine, anesthesiology, geriatrics, surgery, pulmonary and critical care, family medicine and emergency medicine [34], [35], [36], [37], [38], [39], [40], [41]. 

Another recent concept connected with CBME is that of “milestones.” En route to competence, trainees develop progressively in a way that can be defined as stages or performance levels. In the 1980s, Dreyfus and Dreyfus defined five stages in the development of skill: Novice, Advanced Beginner, Competent, Proficient, and Expert [42]. These have been elaborated and applied to the medical domain by Carraccio and colleagues [43]. Note that in this model, “competent” is a threshold stage that could allow for a justified entrustment decision, a stage at which society would accept unsupervised practice by this person [44], [45], and being “competent” certainly does not preclude further development toward proficiency and expertise. The USA Accreditation Council for Graduate Medical Education has built their “next accreditation system” on a foundation of milestones [46], defined as “developmentally based, specialty specific achievements that residents are expected to demonstrate at established intervals as they progress through training.” 

## Speculating about future developments

Postgraduate medical education is in transition. Much has happened since the turn of the century in the USA, Canada and other countries. While Competency-based medical education also meets with criticism [47], [48], it remains a popular concept that continuously evolves [21], [49], [50] and that likely will determine the near future of postgraduate medical training around the world. With the continued pursuit of graduating medical specialists for unsupervised practice who meet predefined standards [51], time flexibility will ask for a flexibility, and hence adaptation of legislation. Postgraduate programs now have a fixed length, and CBME-variability, even if benefits for quality and safety of patient care can be established, will require major efforts in organizational and regulatory sense. Flexible training arrangements will also be necessary to accommodate the education of clinician researchers and to better accommodate family planning than is currently possible. The debate on reasonable and effective working hours for postgraduate training will likely lead to a further decrease in the 21^st^ century [52], [53].

Another issue that will ask attention is the continuum of medical training. While a century ago the basic medical degree was sufficient for independent practice of most medical trainees, now it has become embedded in a continuum [54]. Continuum-programs are currently being explored [55] and it is very well possible that the strict divide between undergraduate and postgraduate training will disappear to a great extent. At least the continued increase of training length before unsupervised practice, which has doubled across one century, cannot be sustained in the future. 

## Notes

This article is based to a large extent on Ten Cate O, “Medical Education, Competency-Based” in the Wiley Blackwell Encyclopedia of Health Illness, Behavior, and Society by Cockerham WC, Dingwall R and Quah, SR (Eds), 2014 John Wiley & Sons, Ltd (pp 1329-1335). Permission was obtained to republish this entry.

## Competing interests

The author declares that he has no competing interests.
